# Enhanced rock recognition via EVSS-integrated YOLO11: A deep learning approach for precise geological classification

**DOI:** 10.1371/journal.pone.0341862

**Published:** 2026-04-24

**Authors:** Fei Zhao, Xiaopeng Leng, Ming Zhu, Sijia Luo, Jian Huang

**Affiliations:** 1 State Key Laboratory of Geohazard Prevention and Geoenvironment Protection, Chengdu University of Technology, Chengdu, China; 2 College of Computer Science and Cyber Security, Chengdu University of Technology, Chengdu, China; 3 BIM Technology Application Industry Research Center, Sichuan Communication Surveying and Design Institute, Chengdu, China; Ministry of Education, MOROCCO

## Abstract

Rock identification plays a fundamental role in geological work, particularly in resource reservoir characterization, stratigraphic division, engineering stability assessment, and hazard prevention. However, traditional manual identification approaches exhibit low efficiency and limited ability to capture dynamic and fine-grained features. To address these challenges, this study employs image recognition and object detection techniques to classify igneous, sedimentary, and metamorphic rocks. We propose an improved You Only Look Once version 11 (YOLO11)-based model by integrating the Efficient Visual State Space (EVSS) module, which enhances the extraction of key rock characteristics—such as texture and fractures—by modeling long-range spatial dependencies and overcoming the locality limitations of conventional convolutional networks. The proposed method is evaluated against three mainstream deep learning models. Experimental results show that the EVSS-enhanced YOLO11 achieves the highest classification accuracy of 92%, outperforming the Vision Transformer (ViT, 85%), ResNet (74%), and the standard YOLO11 (87%). In object detection tasks, the EVSS-integrated YOLO11 also demonstrates superior performance, achieving a mean average precision at 50% intersection-over-union (mAP50) of 91.8% compared to 87.7% for the original YOLO11. By combining efficient visual feature modeling with multi-scale detection capability, this study confirms the effectiveness and robustness of the EVSS-YOLO11 framework for rock image identification, providing strong technical support for intelligent geological analysis.

## Introduction

China is endowed with abundant geological resources and diverse geological formations, in which rocks and minerals have traditionally served as fundamental objects of geological research [1]. Accurate rock identification plays a critical role in resource evaluation, stratigraphic interpretation, engineering stability assessment, and geohazard prevention. Conventional identification techniques, including hand specimen observation, thin-section petrography, elemental analysis, and mineralogical testing, remain authoritative but are often constrained by laboratory dependence, instrument precision, and expert experience [2]. These limitations significantly restrict efficiency and scalability under field conditions, motivating the development of automated and intelligent lithology identification methods. Recent studies have further highlighted that improving robustness and deployability under realistic field environments remains a central challenge for refined lithology identification [3].

With the rapid advancement of graphics processing unit (GPU) computing, deep learning has emerged as a dominant paradigm for automated rock identification. Image-based approaches, in particular, have attracted increasing attention due to their non-destructive nature, low acquisition cost, and suitability for large-scale deployment. Early studies demonstrated that convolutional neural networks (CNNs) could effectively extract discriminative features from rock images for lithological classification [4,5]. Subsequent research explored deeper architectures and transfer learning strategies, such as Inception-based and ResNet-based models, to further enhance classification accuracy [6; Alzubaidi et al., 2021]. Comparative studies on rock microscopic and core images have confirmed that CNN-based models consistently outperform traditional machine learning approaches in feature representation and classification performance [7,8,9].

Despite these advances, conventional CNN-based classification models are inherently limited by their local receptive fields, which restrict their ability to capture long-range spatial dependencies and global texture patterns characteristic of complex rock surfaces. To address this limitation, more recent studies have introduced global modeling mechanisms, including attention-based networks and operator-learning frameworks. For example, Marques et al. [11] proposed a lithological classification model based on Fourier neural operators combined with channel-wise self-attention, achieving improved global feature representation. Lightweight CNN architectures have also been explored to balance accuracy and computational efficiency, such as RockDNet and pruning-based optimization strategies for intelligent lithology identification [10,3]. However, many of these models still incur considerable computational overhead or require large-scale annotated datasets, posing challenges for deployment in real-time or resource-constrained geological applications.

Beyond pure image classification, object detection frameworks have emerged as an effective alternative for rock recognition by explicitly localizing rock regions and suppressing background interference. Xu et al. [13] demonstrated that Faster R-CNN–based architectures can achieve high detection accuracy for rock images, while subsequent studies further validated the effectiveness of object detection pipelines in lithology identification tasks [13,19]. Compared with image-level classification, object detection enables more robust feature learning under complex backgrounds by focusing on target rock regions, which is particularly advantageous for field-acquired imagery with cluttered environments.

Among object detection methods, the You Only Look Once (YOLO) family has gained widespread adoption due to its favorable balance between detection accuracy and inference efficiency. YOLO formulates object detection as a unified regression problem, enabling end-to-end training and real-time performance [16]. Recent lightweight variants further improve adaptability to variable object scales and complex textures, making them suitable for geological imagery. Nevertheless, existing YOLO-based approaches still face challenges in capturing global texture dependencies and multi-scale structural features inherent to rock images, especially under diverse lighting and weather conditions encountered in field surveys.

Therefore, the objective of this study is to develop an efficient and robust rock recognition framework by integrating the Efficient Visual State Space (EVSS) module into the YOLO11 architecture. The proposed method aims to enhance global spatial dependency modeling and texture representation while preserving the lightweight inference characteristics of YOLO11. A ten-class rock image dataset covering igneous, sedimentary, and metamorphic lithologies is constructed to support systematic evaluation. Through comprehensive experiments on both image classification and object detection tasks, this work seeks to provide a practical and deployable solution for intelligent lithology identification under realistic geological imaging conditions.

The dataset and trained models supporting the findings of this study are based on a self-constructed dataset developed by the authors and are publicly available on Figshare at https://figshare.com/articles/dataset/__/30608453

## Dataset description

**Fig 1:**
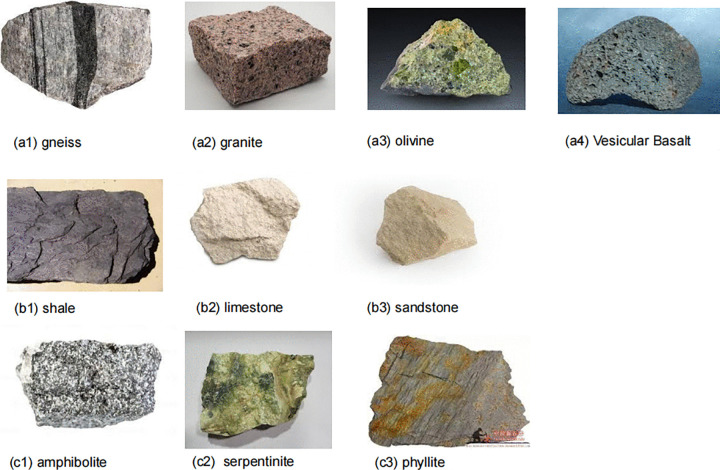
Typical examples of rock image dataset. Representative samples from the ten-class rock dataset spanning igneous, sedimentary, and metamorphic lithologies.

**Fig 2:**
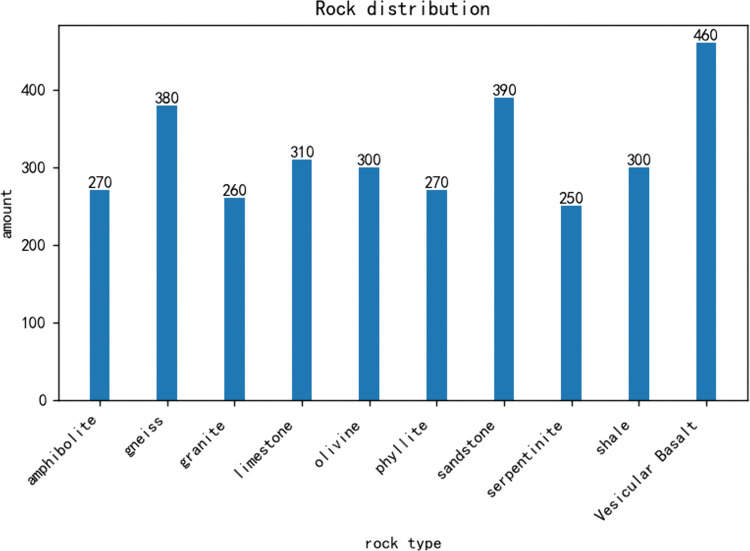
Statistical chart of dataset quantity. Bar chart showing the number of images per class in the constructed dataset. The distribution reflects natural class imbalance, which was considered during training and addressed with data augmentation.

The experimental dataset consists of 10 common rock types, comprising a total of 3,190 images. Example images of various rock types are shown in Fig 1. The dataset includes three major rock categories: igneous rocks (e.g., granite, basalt), sedimentary rocks (e.g., sandstone, shale), and metamorphic rocks (e.g., amphilbolite, phyllite), representing the primary rock types encountered in geological surveys. The composition of the dataset is presented in Fig 2.

**Fig 3:**
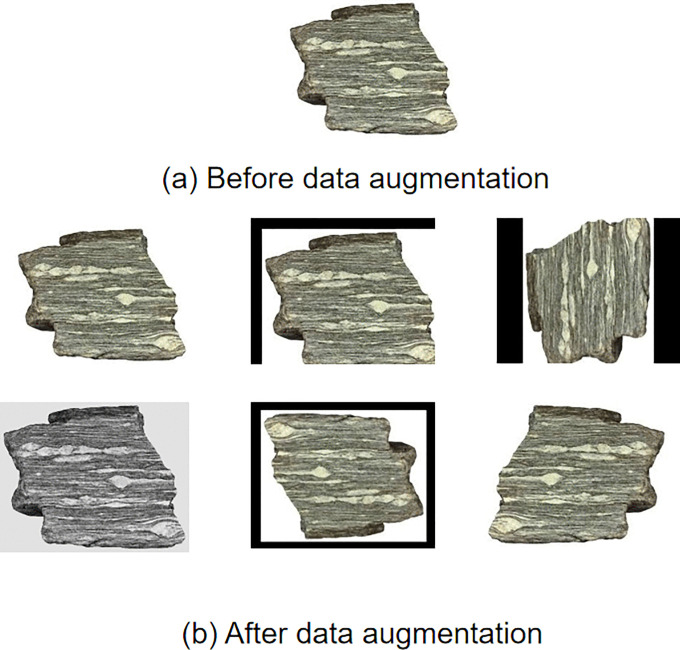
Examples of augmented rock images. Samples generated through data augmentation operations, including rotation, brightness adjustment, flipping, and cropping. These augmentations increase data diversity and improve model robustness against variations in imaging conditions.

During the data preparation stage, systematic data augmentation was applied to the original samples, which included the following techniques: geometric transformations (scaling, panning, rotation), image flipping, photometric adjustments (simulating different lighting conditions), and spatial transformations, among others. These enhancements increased data diversity, enabling the model to better adapt to varying imaging conditions encountered in practical applications. Some of the augmented datasets are shown in Fig 3.

To ensure the stability and generalization capability of the model, the dataset was restructured into three subsets following a 7:2:1 split. Specifically, 70% of the images were allocated for training to support model learning and parameter optimization, 20% were used as a validation set to guide hyperparameter tuning and performance monitoring during training, and the remaining 10% were held out as an independent test set to provide an unbiased assessment of the final model’s generalization performance.

## Algorithmic framework

### YOLO11 network architecture

Rock images typically exhibit multi-scale textures, irregular edges, and complex background clutter, presenting dual demands on detection models for both local detail retention and global context modelling capabilities. YOLOv11, with its dynamic feature fusion mechanism and lightweight design, demonstrates excellent multi-scale object adaptability while maintaining high inference speed, making it the ideal foundational architecture for this study.

**Fig 4:**
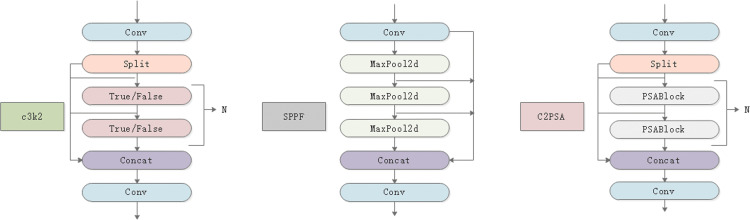
Detailed structure of YOLO11 section. Schematic illustrating the main modules of the YOLO11 architecture used in this study. The figure highlights the feature extraction layers, spatial pyramid pooling, and detection head responsible for multi-scale prediction.

YOLO11 builds upon YOLOv8 and incorporates features from all previous YOLO versions. Compared to YOLOv8, it introduces several new modules, including the C3k2 block, Spatial Pyramid Pooling Fusion (SPPF), and Channel Pixel Spatial Attention (C2PSA), which significantly enhance its feature extraction capabilities and improve multi-scale target recognition accuracy. The structure of each module is illustrated in Fig 4.

Compared to YOLOv8, YOLO11 introduces the C3K2 module, a custom implementation of the Cross Stage Partial (CSP) Bottleneck, which balances speed and performance by utilizing two smaller convolutional kernels. The C3K2 module includes a parameter that determines whether to activate it; if set to false, the module reverts to the version used in YOLOv8, the C2f module. The decision to use this module depends on the training scenario: for larger model versions (M/L/X), the value is set to true, while for smaller versions (N/S), it is set to false. YOLO11 retains the original SPPF module, which, compared to the traditional SPP module, employs fixed-size pooling kernels to perform repeated maximum pooling, significantly reducing computational costs. Following SPPF, the C2PSA module is introduced, enhancing spatial attention in the feature map and improving the model’s focus on important regions of the image. By pooling spatial features, the model is able to more efficiently concentrate on regions of interest. YOLO11 substantially improves feature extraction by adopting an optimized backbone and neck architectures, leading to enhanced object detection accuracy. Its refined design and optimized training flow not only enable faster processing speeds but also achieve a strong balance between accuracy and performance. Furthermore, YOLO11 exhibits excellent environmental adaptability, making it suitable for deployment on edge devices, cloud platforms, and NVIDIA GPU-enabled systems.

### Efficient spatial scanning module

**Fig 5:**
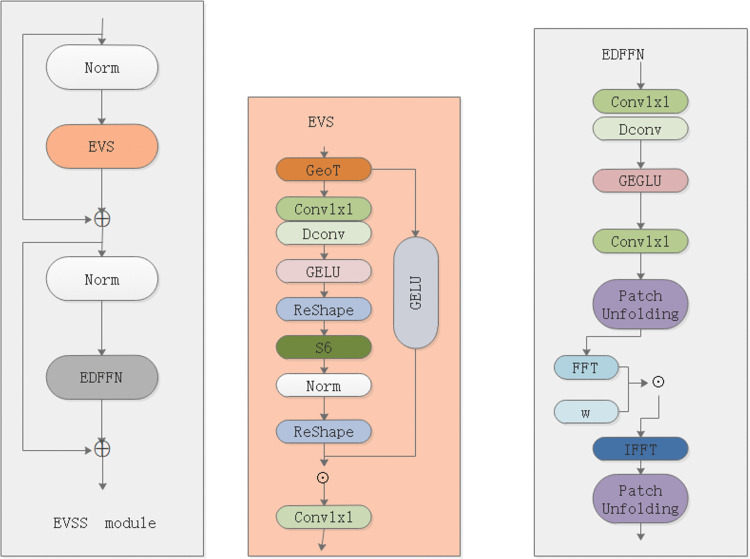
EVSS structure diagram. Diagram of the EVSS feature modeling block integrated into YOLO11. The module enhances long-range dependency modeling and strengthens texture representation through efficient state-space operations.

Rock images are characterized by intricate textures, directional structures (e.g., foliation, bedding, and fracture patterns), and long-range spatial correlations that are critical for accurate lithological discrimination. However, conventional convolutional networks struggle to model such non-local dependencies, while attention-based architectures often incur prohibitive computational overhead—particularly problematic for field-deployable systems. To address this, we integrate the Efficient Visual State Space (EVSS) module into YOLO11. The EVSS is a state-space modeling structure originally developed for visual tasks, which effectively reconciles global context modeling with fine-grained local detail preservation. Specifically, it incorporates a geometric transformation-guided spatial perception mechanism, a selective state scanning strategy, and a frequency-domain-enhanced feed-forward network co-optimization, yielding a lightweight yet structurally aware framework. As illustrated in Fig 5, this design enables the model to capture orientation-sensitive geological features across scales without significant latency increase—making it particularly suitable for rock image analysis.

The geometry-guided scanning mechanism breaks the spatial limitation caused by serialization after image flattening by introducing lightweight geometric transformations (e.g., transpose and flip) prior to the state scanning, enabling the model to achieve multi-direction perception at a very low cost. Let the current state be sₜ and the input be xₜ, the state update formula is as follows:


$$S_{t}=\left(\text{1}-g_{t}\right)*S_{t-\text{1}}+g_{t}*Update(X_{t})$$ (1)

The selective state scanning path is inspired by Mamba’s Selective Scan (S6) mechanism, which dynamically selects the state update path based on the input content, alleviating the issue of timing dependency destruction caused by image spreading. In EVSS, state scanning is no longer performed in a fixed direction; instead, the scanning order is selectively adjusted based on the content of the current feature map. The state update and spatial response are then fused using a Gating Mechanism:


Y=g*Update(X)+(1−g)*X (2)

The EDFFN (Enhanced Dual-path Feed Forward Network), as a companion module of EVSS, further enhances frequency-domain expressiveness and structural alignment stability. The input image is divided into multiple patches, with a frequency-domain transform (e.g., fast Fourier transform (FFT)) or local attention operation applied to each patch. All patches are then spliced together, and the original image size is restored to achieve global consistency.

The introduction of the Efficient Visual State Space (EVSS) module in rock recognition primarily addresses the challenge of balancing global-dependent modeling with local feature capture, a limitation faced by traditional models when processing high-resolution geological images. Rock identification requires precise resolution of fine details, such as rock texture and structure, which demands high spatial information extraction capabilities from the model. By applying geometric transformations (e.g., flip and transpose) before the state-space model (SSM), EVSS effectively captures non-local spatial information without significantly increasing computational complexity. This approach replaces the traditional multi-directional scanning strategy, avoiding a sharp rise in computational costs. Not only does it retain the advantage of SSM in handling long-range dependencies, but it also enhances the model’s ability to understand the spatial structure of geological images, improving both the accuracy and efficiency of rock identification—particularly when dealing with complex, high-resolution image data. Thus, the EVSS module serves as a powerful tool for rock recognition, enhancing the quality of geological data analysis.

### YOLO11 incorporating EVSS

**Fig 6:**
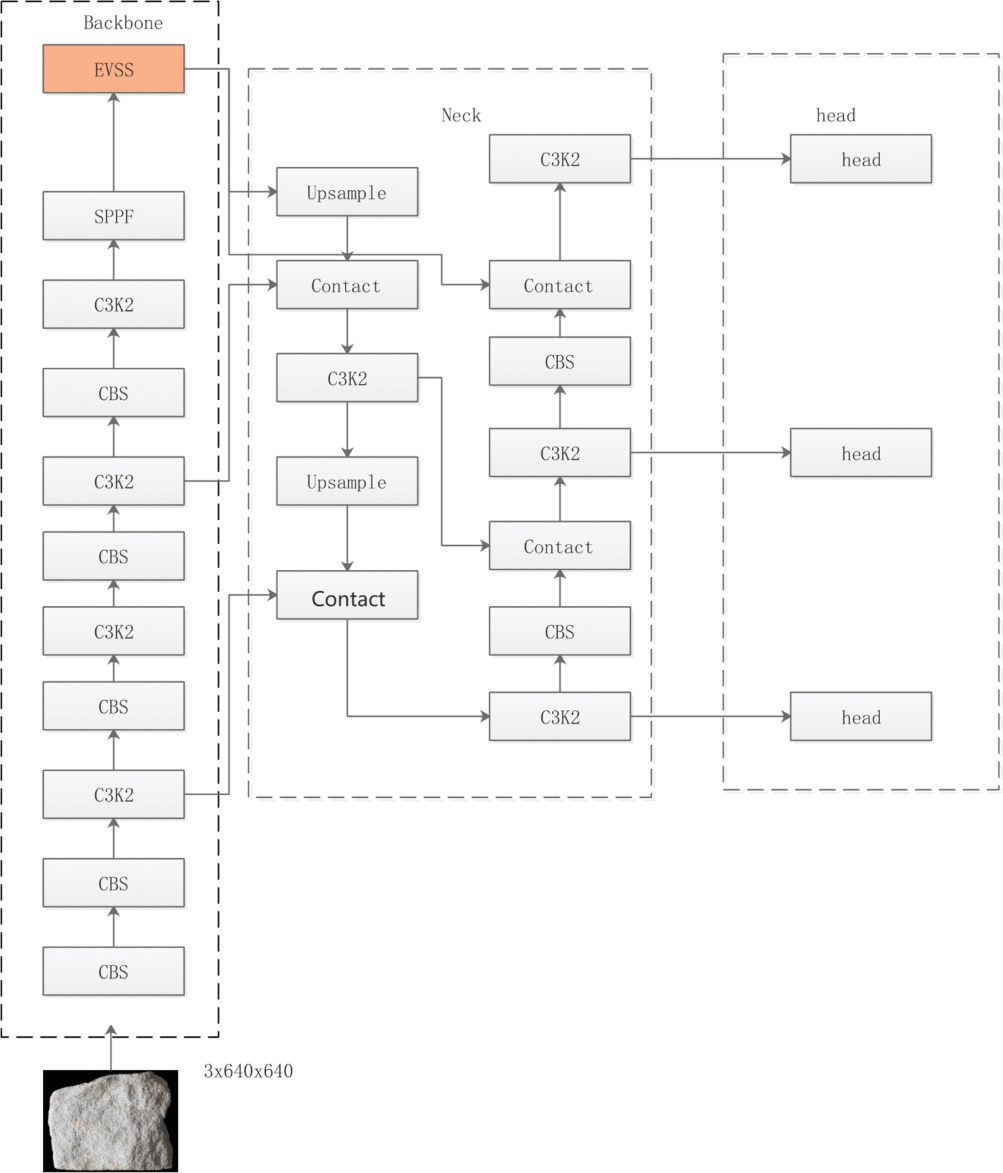
YOLO11 network architecture with EVSS convergence. Integration of the EVSS module within the YOLO11 framework. The diagram shows where EVSS is incorporated to improve feature extraction while maintaining lightweight inference.

The new C2PSA module added to YOLO11 is an attention mechanism designed to enhance multi-scale feature processing and improve target detection accuracy. In contrast to C2PSA, which relies on attention mechanisms to extract key features, EVSS more efficiently retains the spatial semantic structure of the image. By introducing geometric transformations (e.g., flipping, transposition) and selective state scanning mechanisms, EVSS enhances the model’s ability to capture non-local spatial information without significantly increasing computational effort. Consequently, the original C2PSA module in YOLO11 is replaced by the EVSS module, resulting in improved performance in both image recognition and target detection experiments. The improved YOLO11 network architecture, incorporating EVSS, is illustrated in Fig 6.

The input image is processed through multiple stages in the YOLO11 architecture. Multi-scale features are first extracted by the Backbone network, followed by feature fusion and enhancement in the Neck, and finally, target detection results at different scales are produced by multiple Heads. In the Backbone network, the input image is progressively downsampled through successive convolutions. Feature enhancement is performed by the C3K2 module, the SPPF module captures global information, and the EVSS module provides global modeling and local structure. The Neck network receives multiple feature maps from the Backbone, restores deeper feature maps to a higher resolution via upsampling, and combines feature maps at different scales using the Contact module. The spliced feature maps are then further enhanced by the C3K2 module. Finally, the Head network refines the features and outputs target detection results at various scales.

## Experiments and analysis of results

### Experimental environment

Python language programming is used to realize the above network architecture, using Pytorch framework, version 2.8.0.1, python version 3.11. The experimental environment is configured as follows: 1 NVIDIA 4090 Ti graphics card (24GB video memory), AMD EPYC 7J13 64-Core Processor CPU, 16-core CPU configuration, 62.9G RAM, 20G system disk and 50GB SSD data disk, supporting the highest CUDA12.4 version.

### Classification experiments

**Fig 7:**
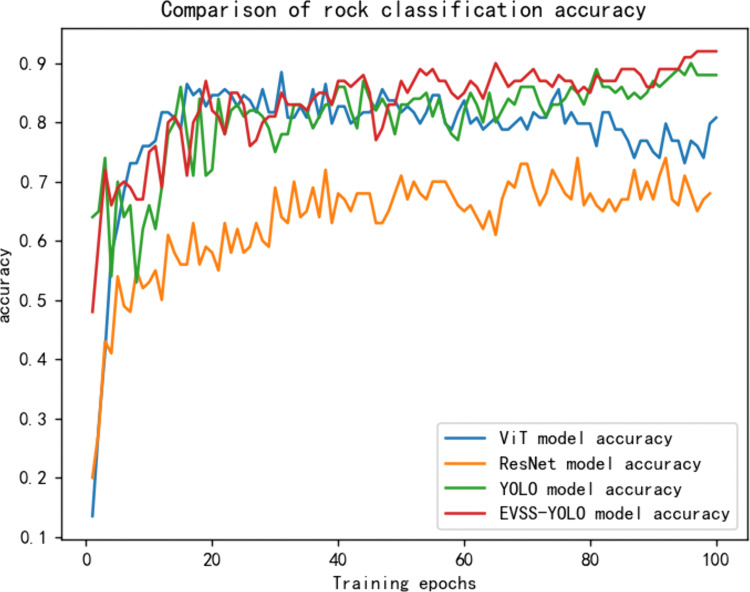
Four model training accuracies. Accuracy trends during training for ResNet, ViT, YOLO11, and the proposed EVSS-YOLO11. The EVSS-enhanced model demonstrates faster convergence and higher final accuracy.

**Fig 8:**
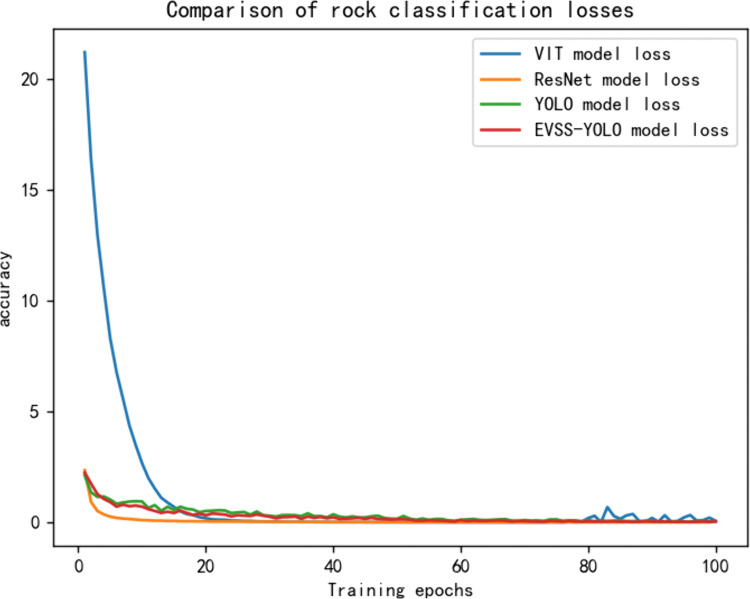
Four model training losses. Loss trajectories for all models throughout the training process. The EVSS-YOLO11 model exhibits more stable optimization and lower final loss values, indicating improved feature learning.

Given that the dataset consists of rock hand specimens and the recognition target occupies a large portion of the image, the experiments prioritize image classification for rock recognition. Four models—ViT, ResNet, YOLO11, and YOLO11 fused with EVSS—are selected for comparison. Figs 7 and 8 illustrate the accuracy and loss of these models after 100 training epochs, respectively. In classification tasks, accuracy measures the proportion of samples correctly predicted by the model, providing an intuitive reflection of its overall recognition capability. Loss (such as cross-entropy loss) quantifies the discrepancy between the model’s predicted distribution and the true labels, indicating the convergence of the optimisation objective. Combining these metrics enables a comprehensive evaluation of model quality across two dimensions: performance and training stability.

The experimental results, shown in Table 1, indicate that as the number of training epochs increases, all four models reach convergence. The accuracy of the ResNet model is initially lower but improves gradually over time. However, the overall accuracy of ResNet fluctuates, ultimately stabilizing around 0.7. The ViT model shows steady improvement in accuracy during the early stages, with significant feature learning optimization evident. The model converges after 30 epochs, with the loss nearing zero; however, accuracy plateaus and does not exceed 90%, possibly due to limitations in model capacity or data complexity. The YOLO11 model exhibits rapid accuracy improvement with strong early learning, converging after around 20 epochs, and achieving a final accuracy of 87%. The EVSS-YOLO model performs the best, consistently improving and reaching a final accuracy of 92%. Overall, the EVSS-YOLO model outperforms the other models in hand specimen rock image classification, followed by YOLO11, ViT, and ResNet, which is slightly inferior in both accuracy and stability. This performance enhancement is reflected not only in improved accuracy but also in the rapid convergence and low final value (0.017) of the training loss. This indicates that the EVSS module effectively mitigates the model’s overfitting to local noise while enhancing its ability to learn intrinsic rock characteristics, such as mineral particle arrangement and fracture distribution.

**Table 1:** Training results of four models for rock recognition.

model	loss	accuracy
**Vit**	0.06	0.85
**ResNet**	0.004	0.72
**Yolo11**	0.046	0.88
**YOLO11 with EVSS**	0.017	0.92

**Fig 9:**
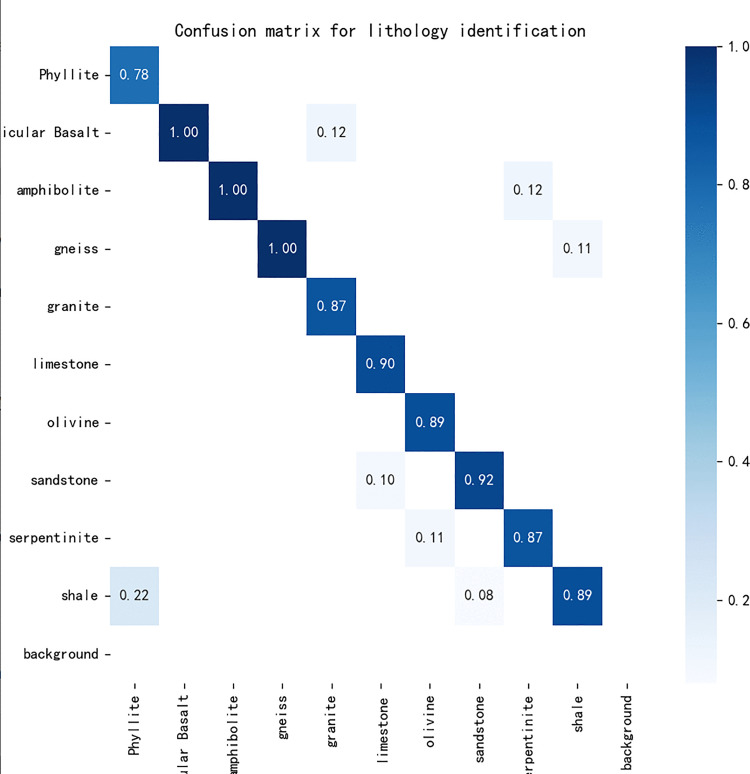
Confusion matrix for classification experiments. Confusion matrix summarizing the model’s classification performance across all ten rock categories. Diagonal elements indicate correct predictions, while off-diagonal entries reveal misclassification patterns.

The confusion matrix is a crucial tool for evaluating the performance of a classification model, illustrating the relationship between predicted classifications and actual labels in matrix form. It enables visualization of classifier performance across different categories. For instance, the diagonal elements represent the proportion of correctly classified samples, while the off-diagonal elements indicate misclassifications. The confusion matrix for the YOLO11 classification experiments using the fused EVSS is shown in Fig 9.

As shown in Fig 9, the model achieves near-perfect classification for rocks with distinctive structural or textural signatures—such as porphyritic basalt (characterized by phenocrysts in a fine matrix), hornblende (strong pleochroism and prismatic habit), and gneiss (pronounced banding)—with accuracies approaching 100%. This confirms the model’s capacity to leverage salient geological features for robust discrimination.

However, notable confusions occur among lithologies with visually overlapping characteristics. Specifically, shale is misclassified as kyanite (22%) and sandstone (8%), likely due to their shared fine-grained appearance and low-contrast surface textures under natural lighting. Similarly, serpentine and peridotite—both greenish, massive ultramafic rocks—exhibit an 11% mutual confusion, reflecting their mineralogical proximity and similar weathering behavior. The 11% misclassification between sandstone and limestone further underscores the challenge posed by carbonate-cemented clastic rocks, which can mimic pure carbonate textures in RGB imagery.

These patterns reveal a fundamental constraint of purely vision-based lithology identification: **rocks with convergent surface morphology but divergent origins may remain ambiguous without additional modalities (e.g., spectral response or hardness cues)**. Future work will explore multi-modal fusion to resolve such ambiguities, particularly for fine-grained or altered rock suites like shale, mylonite, and serpentine.

### Target detection

Due to the complexity of the application scenario, the detection images cannot achieve the same clarity in rock features and background as the rock hand specimens. Therefore, the later part of the experiment uses the YOLO11 model, which performed better in the classification experiments, for target detection. While image classification assigns labels to the entire image, target detection not only identifies object categories but also locates each object within the image. In the context of rock recognition, target detection technology effectively addresses the problem of inaccurate recognition results caused by background clutter in real-world scenarios.

The original dataset is annotated by drawing bounding boxes using MakeSense, and corresponding annotation files are generated for each image. The dataset is then divided into training, validation, and test sets with a 7:2:1 ratio. The YOLO11 model is trained on this dataset, with the EVSS module added to enhance model accuracy.

The experimental results are shown in Table 2, and the evaluation metrics include precision: a measure of the accuracy of the model’s prediction, i.e., the proportion of all samples predicted to be positive cases that are actually positive cases; recall: a measure of the coverage of the model’s detection, i.e., the proportion of all real targets that are correctly detected; and mAP0.5: 0.95 (B): the average of the computed mAPs over the range of the IOU thresholds from 0.5 to 0.95.

**Table 2:** Experimental results of YOLO11 with YOLO11 fused with EVSS.

model	Accuracy	recall	mAP@50	mAP@50–95
**YOLO11**	0.736	0.79	0.877	0.784
**YOLO11 with EVSS**	0.825	0.804	0.918	0.828

**Fig 10:**
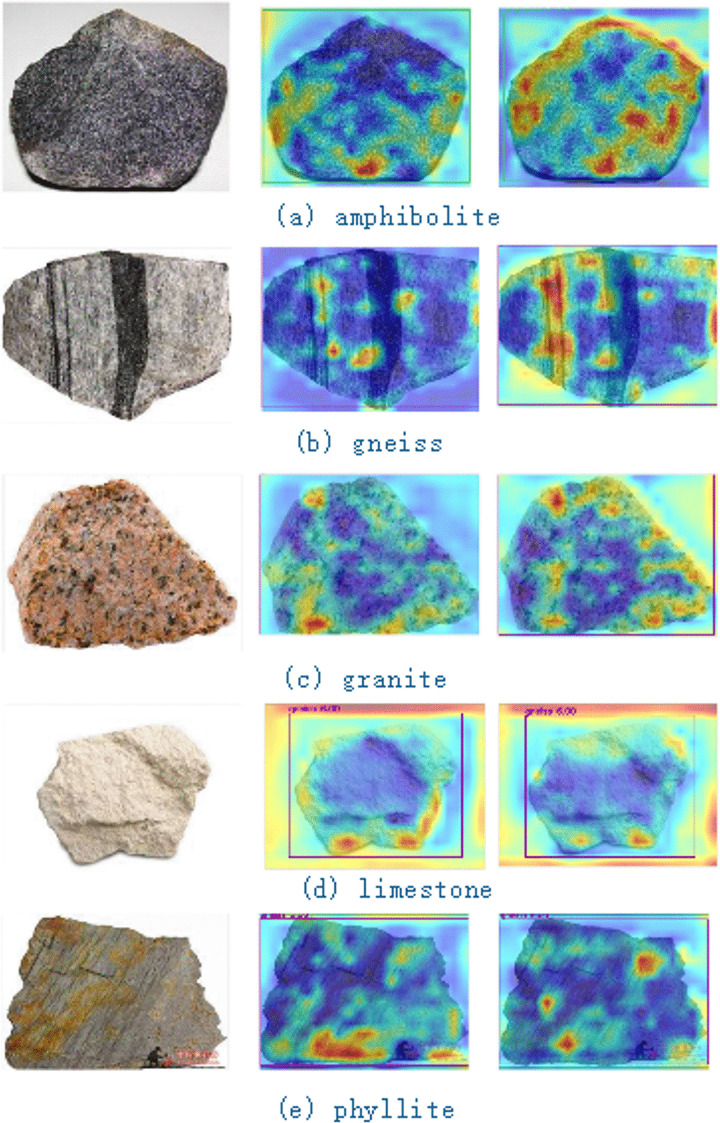
Comparison of Grad-CAM heat maps across models. Visualization of attention distribution generated by different models. The EVSS-YOLO11 heat maps show strengthened focus on key mineral textures and structural features compared to baseline models.

The experimental results show that YOLO11 fused with EVSS is comprehensively better than YOLO11, with improvements in all four metrics: precision, recall, mAP@50 and mAP@50–95, indicating that the improved model performs better in the rock recognition task. The recall rate is improved from 73.6% to 82.5%, and the probability of missed detection is greatly reduced by adding the efficient spatial scanning module, which can detect more rock targets. mAP@50 and mAP@50–95 are both improved, which shows that the improved model not only has a better detection capability, but also improves the localization accuracy. comparison of the heat map of some rock categories of Yolo11 and Yolo11 with the fusion of EVSS is shown in Fig 10. On the left is a picture of the rocks used for testing, in the center is the thermal map of yolo11, and on the right is the thermal map of YOLO11 fused with EVSS.

Although the EVSS-enhanced YOLO11 model achieves a high mAP@50 of 0.918, the mAP@50–95 decreases to 0.828. This gap reflects an important characteristic of the detection task rather than a degradation in model quality. The higher mAP@50 indicates that the model is highly effective at identifying the presence and approximate location of rock targets. However, the stricter IoU thresholds in mAP@50–95 require precise alignment between predicted and ground-truth bounding boxes.

Rock objects in field imagery often exhibit irregular geometries, indistinct boundaries, heterogeneous textures, and partially occluded surfaces, which introduce ambiguity in defining the exact spatial extent of the target. As a result, even small deviations in bounding box placement—such as conservative cropping caused by background clutter—can lead to a notable performance drop at higher IoU thresholds. This trend is commonly observed in detection tasks involving non-rigid or morphologically complex objects.

Despite the reduced score under stricter IoU constraints, the EVSS-YOLO11 model consistently produces stable bounding boxes and maintains high detection reliability. The spatial context modeling capability introduced by EVSS enables the model to accurately capture rock regions even when fine-grained boundary alignment is challenging. Therefore, the mAP gap primarily reflects the inherent complexity of geological targets rather than a limitation of the model itself.

As shown in Fig 10, YOLO11 fused with EVSS significantly improves thermogram performance compared to the original YOLO11 model. Specifically, when detecting rock categories, the thermogram of the original YOLO11 model exhibits weak responses in certain rock edge regions, resulting in false and missed detections. In contrast, the heat map from the YOLO11 model fused with EVSS shows clearer boundaries for the rock targets, stronger overall response, and a notable reduction in both misdetections and missed detections. This demonstrates that the addition of the EVSS module enhances the model’s ability to detect rock category features, improving both detection accuracy and robustness. These experimental results underscore the effectiveness and superiority of the improved method in rock image detection.

**Fig 11:**
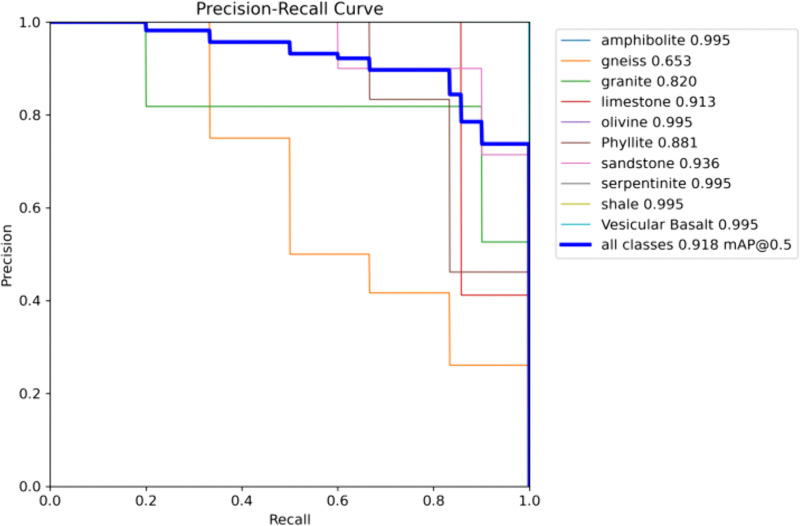
Precision–Recall (PR) curves for the detection experiment. PR curves illustrating detection performance across IoU thresholds. The EVSS-YOLO11 model achieves higher area under the curve (AUC), reflecting better precision–recall balance.

The results of YOLO11 with fused EVSS on the training set are shown in Table 3, and the corresponding precision–recall (PR) curves are presented in Fig 11. The YOLO11 model fused with EVSS performs well in the classification of ten rock types, achieving a high overall mAP@50 (mean accuracy with an IoU threshold of 50%). However, there are notable differences in the recognition performance across categories. Specifically, the model achieves very high accuracy in classifying hornblende, peridotite, serpentine, shale, and stromatolite basalt, with mAP@50 values all reaching 0.995. This suggests that the visual features of these rocks—such as texture, color, or structure—are well differentiated in the dataset, allowing the model to effectively capture their key characteristics.

**Table 3:** Training results of YOLO11 rock recognition fused with EVSS.

category	mAP@50
**amphibolite**	0.995
**gneiss**	0.653
**granite**	0.82
**limestone**	0.913
**olivine**	0.995
**phyllite**	0.881
**sandstone**	0.936
**serpentinite**	0.996
**shale**	0.995
**Vesicular Basalt**	0.995

The accuracy for limestone, sandstone, and millimetre rock is slightly lower, ranging from 0.881 to 0.936, indicating that the model has adequately learned their features, though there may be some misdetections or omissions. In contrast, the mAP@50 for gneiss is only 0.653, which is significantly lower than that of the other categories. This may be due to the complex and variable texture of gneiss (e.g., banded structure) or challenges in generalizing the model due to insufficient samples and inconsistent labeling in the training data. Granite, with a moderate precision of 0.82, may be affected by the diversity in its mineral composition, such as variations in particle size.

**Fig 12:**
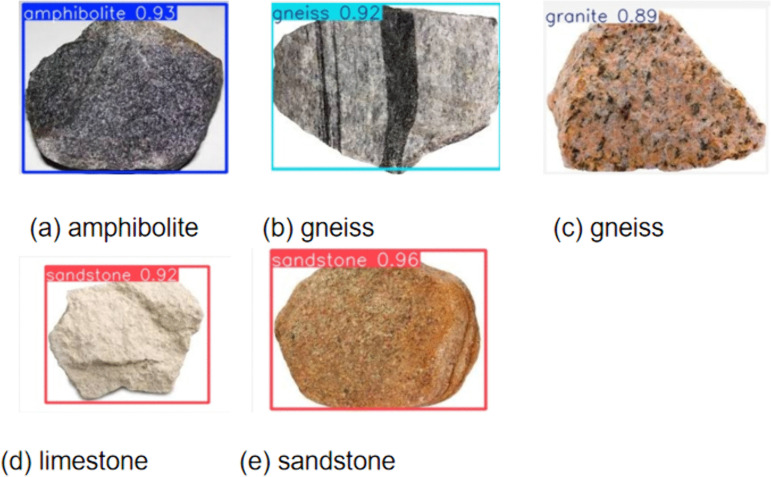
Qualitative results of detection and classification predictions. Examples of model predictions on test images, including bounding boxes, confidence scores, and class labels. The results demonstrate the model’s ability to identify multiple rock types under varying conditions.

The EVSS-enhanced YOLO11 model demonstrates outstanding performance in rock identification, surpassing traditional methods in overall prediction accuracy. As shown in Fig 12, the model consistently achieves high recognition accuracy across various rock types, particularly for layered rocks such as diabase and diorite, with confidence levels exceeding 0.92. This highlights the effective feature extraction capabilities of the EVSS module. Although the model’s precision is slightly lower for structurally complex rocks like granite, it still maintains stable performance. The test results closely align with the mAP metrics from the training phase, confirming the model’s strong generalization ability. In summary, the integration of EVSS significantly improves the accuracy and robustness of rock identification under complex geological conditions, making it highly suitable for practical applications.

## Discussion

The results of this study demonstrate that the integration of the Efficient Visual State Space (EVSS) module into the YOLO11 framework substantially enhances the model’s capability for rock image recognition across both classification and detection tasks. The improved model achieves **92%** classification accuracy and **0.918 / 0.828** mAP@50 / mAP@50–95 in object detection, which indicates strong performance under realistic geological imaging conditions.

### Interpretation of classification performance

Although the classification accuracy of 92% may appear lower than the 98–99% accuracy reported by some earlier studies employing Inception-v3 or 1D-CNN architectures, these prior works typically used laboratory-controlled datasets with limited class diversity and minimal textural variability. In contrast, the dataset used in this study includes ten lithological categories spanning igneous, sedimentary, and metamorphic rocks, each exhibiting substantial differences in color, mineral composition, grain structure, and weathering degree.

Therefore, direct numerical comparison across different datasets is not scientifically appropriate. Within this more challenging and realistic setting, the EVSS-YOLO11 model shows stronger robustness than ViT, ResNet, and the standard YOLO11 baseline, demonstrating that the proposed architecture is effective for multi-class rock recognition under field-like conditions.

### Comparison with previous studies

Prior studies integrating CNNs, Transformer variants, or hybrid architectures have demonstrated effectiveness for lithological classification, but they often require large-scale datasets or controlled environments. Transformer-based models, while strong in global representation, tend to be computationally expensive and less suitable for deployment.

Compared with these approaches, the proposed EVSS-YOLO11 model provides a balanced solution, enhancing global contextual modeling through EVSS while preserving the efficiency of YOLO11’s lightweight architecture. This enables the model to achieve competitive accuracy and detection performance with significantly lower computational overhead.

### Strengths, limitations, and implications

A key strength of this study is the integration of EVSS, which enhances the model’s ability to capture both long-range spatial dependencies and fine-grained textures—critical features for rock recognition. The use of object detection also reduces background interference, improving reliability in complex environments.

However, the model exhibits some limitations:

(1) precise boundary localization remains challenging for irregular rock shapes;
(2) performance may decrease under extreme lighting variation or severe weathering;
(3) the model has not yet been evaluated on hyperspectral or thin-section imagery, which represent other important geological modalities.

Despite these limitations, the results suggest that EVSS-YOLO11 is a promising candidate for practical rock identification systems in field-based geological surveys or mobile platforms.

### Directions for future research

Future work may explore:

(1) integrating geometric priors or boundary-aware loss functions to improve high-IoU localization;
(2) extending the model to multimodal datasets, including hyperspectral or petrographic thin-section images;
(3) developing lightweight variants for real-time deployment on handheld or UAV-mounted devices;
(4) constructing larger, more diverse datasets to enhance generalizability across regions and lithological contexts.

## Conclusion

To address the challenge of low detection accuracy for visually complex rock categories, this study integrates the Efficient Visual State Space (EVSS) module into the YOLO11 architecture, yielding a lightweight yet context-aware rock recognition framework. The proposed model achieves an overall mAP@50 of 92% across ten lithological classes. Notably, rock types with distinctive textural or structural regularities—such as hornblende, peridotite, serpentine, shale, and stromatolitic basalt—attain detection accuracies exceeding 99.5%, indicating that the EVSS-enhanced feature representation effectively captures geologically salient patterns.

These results suggest that incorporating state-space modeling into object detection can better reconcile local detail preservation with long-range spatial dependency learning—a critical requirement for distinguishing fine-grained or structurally homogeneous rocks. While the model demonstrates strong performance on rocks with uniform textures, its relatively lower accuracy on visually ambiguous pairs [12,14,15,17,18,20,21] (e.g., shale vs. kyanite) highlights the inherent limitations of RGB-only analysis. Nevertheless, this work establishes a viable pathway toward real-time, high-accuracy lithological identification in field-deployable systems, and underscores the potential of hybrid CNN–state-space architectures for earth science vision tasks.
